# Bacterial aggregation facilitates internalin-mediated invasion of *Listeria monocytogenes*

**DOI:** 10.3389/fcimb.2024.1411124

**Published:** 2024-07-09

**Authors:** Liam Feltham, Josephine Moran, Marie Goldrick, Elizabeth Lord, David G. Spiller, Jennifer S. Cavet, Mark Muldoon, Ian. S. Roberts, Pawel Paszek

**Affiliations:** ^1^ School of Biology, Faculty of Biology, Medicine and Health, University of Manchester, Manchester Academic Health Science Centre, Manchester, United Kingdom; ^2^ Department of Mathematics, University of Manchester, Manchester, United Kingdom; ^3^ Institute of Fundamental Technological Research, Polish Academy of Sciences, Warsaw, Poland

**Keywords:** *Listeria monocytogenes*, host-pathogen interactions, aggregation, PrfA regulon, live-cell microscopy

## Abstract

Dissemination of food-borne *L. monocytogenes* in the host relies on internalin-mediated invasion, but the underlying invasion strategies remain elusive. Here we use live-cell microscopy to follow single cell interactions between individual human cells and *L. monocytogenes* and elucidate mechanisms associated with internalin B (InlB)-mediated invasion. We demonstrate that whilst a replicative invasion of nonphagocytic cells is a rare event even at high multiplicities of invasion, *L. monocytogenes* overcomes this by utilising a strategy relaying on PrfA-mediated ActA-based aggregation. We show that *L. monocytogenes* forms aggregates in extracellular host cell environment, which promote approximately 5-fold more host cell adhesions than the non-aggregating actA-*Δ*C mutant (which lacks the C-terminus coding region), with the adhering bacteria inducing 3-fold more intracellular invasions. Aggregation is associated with robust MET tyrosine kinase receptor clustering in the host cells, a hallmark of InlB-mediated invasion, something not observed with the *actA-ΔC* mutant. Finally, we show via RNA-seq analyses that aggregation involves a global adaptive response to host cell environment (including iron depletion), resulting in metabolic changes in *L. monocytogenes* and upregulation of the PrfA virulence regulon. Overall, our analyses provide new mechanistic insights into internalin-mediated host-pathogen interactions of *L. monocytogenes*.

## Introduction

*L. monocytogenes* is a facultative intracellular pathogen that is ubiquitous in the environment. It is responsible for several serious clinical syndromes in both humans and animals with listeriosis being typified by high mortality rates (20-30% in humans) despite antibiotic intervention (Cossart, 2011). Human infections are associated with eating contaminated foods, and globally *L. monocytogenes* accounts for 30% of all fatalities as a consequence of food borne infection (Radoshevich and Cossart, 2018). The transmission of *L. monocytogenes* to humans from the food-chain, the ability of *L. monocytogenes* to cause disease in animals and emerging antibiotic resistance create serious health and food security risks (Escolar et al., 2017).

The biology of *L. monocytogenes* has been widely studied and has been a useful tool in discovering insights into fundamental cell biology (Radoshevich and Cossart, 2018). The potential of *L. monocytogenes* to cause systemic infection depends on the ability to cross the intestinal barrier and disseminate into other tissues (Cossart, 2011). A number of *L. monocytogenes* internalins and other cell surface molecules have been implicated in invasion into a range of non-immune host cells (Banović, 2020). The most important are internalins InlA and InlB that specifically recognise two eukaryotic cell membrane receptors, E-cadherin (Ecad) (Mengaud et al., 1996) and the tyrosine kinase receptor MET (Shen et al., 2000a), respectively. Different cell types have distinctive susceptibilities to MET and Ecad-mediated invasion, which is critical for *L. monocytogenes* dissemination in the host (Radoshevich and Cossart, 2018). MET is typically expressed in the endothelium and epithelium, among other tissue and controls cell migration and growth during embryogenesis, also playing a critical role in tumourigenesis (Pizarro-Cerda et al., 2012). Some cell types, including hepatocytes, endothelial cells as well as cell lines including HeLa rely exclusively on MET-mediated invasion since they lack E-cadherin expression (Dramsi et al., 1995; Shen et al., 2000b; Kalender et al., 2022). InlB has been implicated as playing a key role in dissemination via M-cell and Peyer’s patch invasion in the intestine, crossing the blood brain barrier and as such is critical in the development of life threatening systemic *L. monocytogenes* infections (Chiba et al., 2011; Banović, 2020; Maudet et al., 2022). To induce uptake into host cells, InlB binding leads to MET dimerization and the stimulation of localised exocytosis (Van Ngo et al., 2017) via MAP and protein kinase C-α (PKC-α) (Shen et al., 2000a). While intracellular uptake primarily facilities bacterial replication, InlB was also shown to modulate immune response by blocking specific *L. monocytogenes* immune killing of infected monocytes (Maudet et al., 2022).

The virulence of *L. monocytogenes* is controlled via PrfA, a transcriptional activator from the cyclic AMP receptor protein family (de las Heras et al., 2011). PrfA regulates expression of internalin genes, as well as genes required for phagosome escape (*hly, plcA, plcB*), intracellular motility (*actA*) and adaptation to cytosolic growth (*hpt*), among others (Cossart, 2011). PrfA expression and activation is tightly regulated through multiple feedback mechanisms involving transcriptional and post-transcriptional regulation as well as adaptation to environmental conditions such as pH and temperature (Johansson et al., 2002; Reniere et al., 2015; Radoshevich and Cossart, 2018; Krypotou et al., 2019). The PrfA regulon is expressed robustly upon intracellular invasion, in particular in infected blood samples (Toledo-Arana et al., 2009), however PrfA activation (at least in a subset of bacteria) also occurs without presence of host cells (Guldimann et al., 2017; Moran et al., 2023). A consequence of the latter is PrfA and ActA-mediated aggregation and biofilm formation to promote colonisation and persistence of bacteria in the host (Travier et al., 2013). This involves interactions between bacteria through the ActA C-terminal region and is functionally distinct from ActA-mediated intracellular motility (Travier et al., 2013). However, the function of these aggregates during intracellular invasion has not been fully elucidated.

Here we use time-lapse live-cell microscopy to study the role of *L. monocytogenes* aggregation in the infection process in single cells in real time to understand interactions between bacteria and host cells. We demonstrate that, even at a high multiplicity of infection (MOI) of up to 20 bacteria per host cell, an InlB-dependent replicative infection of human epithelial HeLa and primary human umbilical vein endothelial (HUVEC) cells is a rare event, with <10% host cells becoming infected and harbouring replicative bacteria. We show that *L. monocytogenes* uses extracellular ActA-mediated aggregation to enhance frequency of replicative invasions. This effect is mediated via increased adhesion and intracellular invasion, in part due to more permissive InlB/MET interactions in comparison to non-aggregating bacteria. Finally, using microscopy and transcriptomics analyses, we show that aggregation is induced *in vitro* by signals produced by host cells and involves metabolic reprograming of bacteria. Overall, our data provide new mechanistic insights into internalin-mediated invasion strategies of *L. monocytogenes*.

## Results

### *L. monocytogenes* infection involves formation of extracellular aggregates

**Figure 1:**
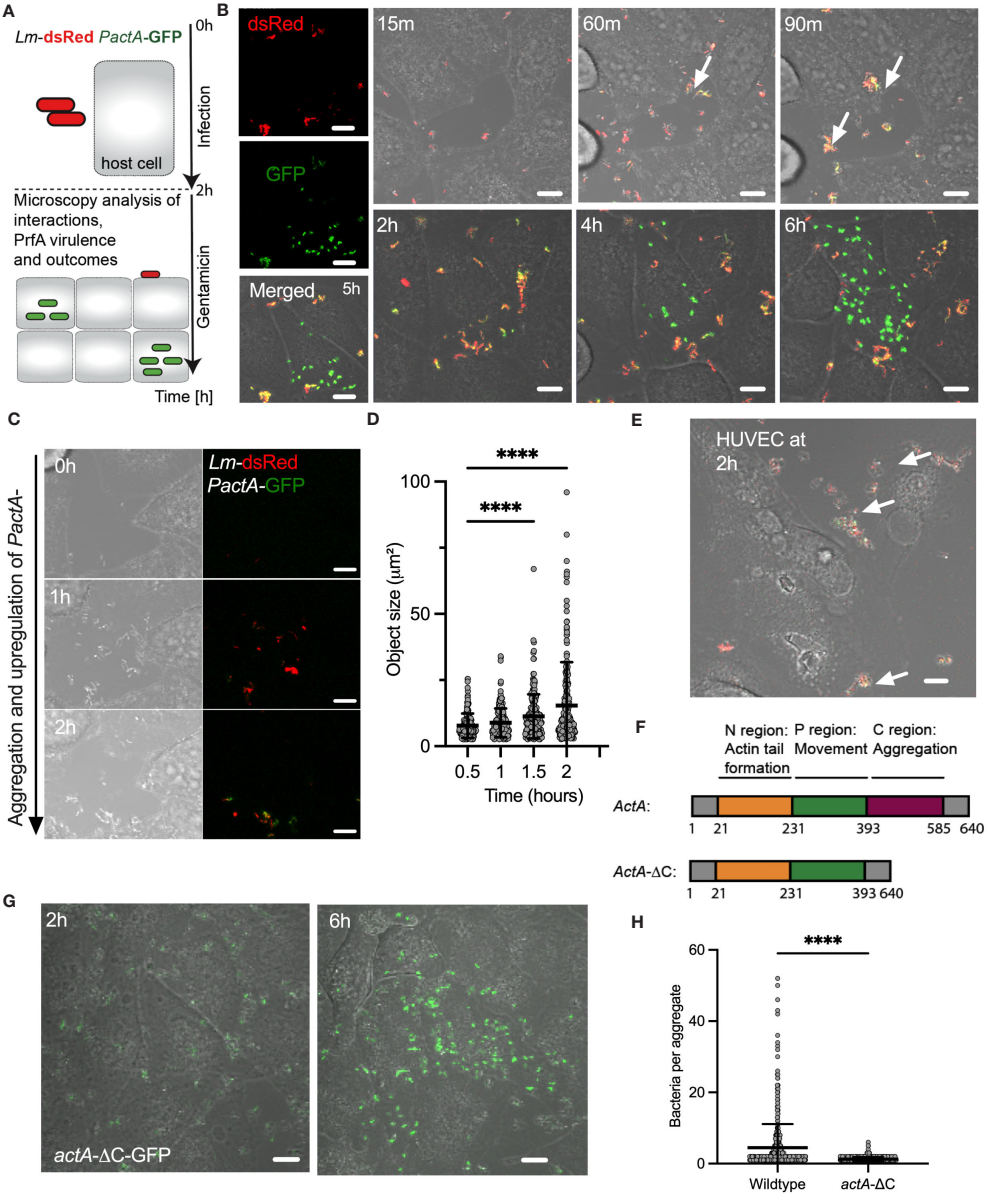
*L. monocytogenes* invasion involves ActA-mediated aggregation. **(A)** Schematic diagram of the live-cell microscopy analyses: Host cells infected with *L. monocytogenes* constitutively expressing dsRed and GFP from integrated *PactA* promoter at MOI=20, incubated for 2 h before washing and adding gentamicin to remove non-adhered and kill extracellular bacteria. Cells are subsequently followed for 6 h with microscopy. **(B)** Representative live-cell microscopy images of replicative invasion events. HeLa cells infected with *Lm-*dsRed *PactA*-GFP reporter strain and visualised for up to 8 h after infection. Shown is a single microscopy field at indicated times, white arrows indicate formation of aggregates. Intracellular replication event highlighted by the robust GFP expression in individual bacteria. On the left are individual (red and green) channels as well as composite including a brightfield image at 5 **(h)** Scale 10 μm. **(C)** Representative live-cell microscopy images of *Lm-*dsRed *PactA*-GFP reporter strain over time as in **(B)**. Scale 10 μm. **(D)** Analysis of aggregate size from data in **(B)**. Shown are estimated aggregate sizes (in μm^2^) based on the dsRed channel at 30, 60, 90 and 120 mins after infection. Shown are 170, 193, 205 and 242 segmented objects based on three independent experiments. Statistical significance (**** = p-value<0.0001) assessed using Kruskal-Wallis test with Dunn’s correction for multiple comparisons. **(E)** Representative confocal microscopy images of aggerate formation upon invasion of primary HUVEC cells. Cells infected with wildtype *Lm*-GFP *PactA*-dsRed at MOI=5. Images representative of three independent experiments at 2 h after infection (before gentamicin treatment). White arrows indicate formation of aggregates. Scale bar 10 μm. **(F)** Schematic representation of full length and AA 393-585 C-terminal deletion of *actA* gene. Different functional domains highlighted in colour. **(G)** Representative microscopy images of HeLa cells infected with *actA-ΔC*-GFP strain at MOI=20. After 2 h gentamicin was added, and the cells were imaged for a further 4 h showing a replicative invasion. Images representative of three independent experiments. Scale bar 10 μm. **(H)** Aggregate size from data wildtype (in B) and *actA*-ΔC (in G). Shown are estimated aggregate sizes (in the number of bacteria) at 2 h post-infection with mean and SD based at least 600 objects from three independent experiments. Statistical significance (**** = p-value<0.0001) assessed using Mann-Whitney test.

To quantify the temporal interactions between *L. monocytogenes* and host cells we used live cell microscopy approaches. We infected HeLa cells, a well-established model of InlB-mediated *L. monocytogenes* infection (Dramsi et al., 1995), with *L. monocytogenes* EDGe: InlA^m^ (Wollert et al., 2007) expressing chromosomally integrated promoter *PactA* driving expression of green fluorescent protein (GFP), in addition to constitutively expressed red fluorescent protein (dsRed), referred herein as *Lm*-dsRed *PactA*-GFP (see Materials and Methods, as well as **Tables 1** and **2** for list of plasmids and strains used in the study). We used a membrane impermeant gentamicin protection assay, with host cells exposed to *L. monocytogenes* for 2 h, before antibiotic treatment, with cells followed by live-cell microscopy for additional 4 h. Based on previous work (Wang et al., 2015), we used MOI of 20 (20:1 pathogen to host cell ratio), which result in spatially separated replicative invasion events and thus allow quantitative investigation of the infection process (**Figure 1A**). We found that changes in MOI were generally associated with intracellular growth, with significantly highest growth at MOI 20 (**Supplementary Figure S1**). Further increase of MOI to 100 resulted in a reduction of growth, consistent with previous reports (Rengarajan et al., 2016).

**Table 1:** List of plasmids used in the study.

Plasmid	Features	Antibiotic	Source
pAD_1_-cGFP	Integrative plasmid with constitutive GFP expression	Cm	(Balestrino et al., 2010)
pAD_3_-*PactA*-GFP	Integrative plasmid expressing GFP under control of P*actA*	Cm	(Balestrino et al., 2010)
pJEBAN6	Plasmid expressing constitutive dsRedExpress	Erm	(Andersen et al., 2006)
pAULA	Cloning vector, pJDC9 derivative, MCS inside *lacZ*, *ori* (Ts)	Erm	(Chakraborty et al., 1992)
pAULA- Δ*inlB*	pAULA vector containing 300bp flanking each side of *inlB*	Erm	This study
pAULA-*actA-ΔC*	pAULA vector containing 300bp flanking each side of *actA* and *actA* gene with deletion of nucleotides 1324 – 1824	Erm	This study

**Table 2:** List of *L. monocytogenes* strains used in the study.

Bacterial strain	Features	Source
*L. monocytogenes* EGDe::InlA^M^	Wildtype, serotype 1/2a. Murinised InlA protein. Used as background strain for all strains in this study	(Wollert et al., 2007)
*L. monocytogenes* EGDe::InlA^m^::*actA*-ΔC	EGDe::InlA^m^ with partial deletion of *actA* gene (C-terminal region) of EGDe, deletion of nucleotides 693-1179.	(Travier et al., 2013), redeveloped in this study
*L. monocytogenes* EGDe::InlA^m^::GFP (Lm-GFP)	EGDe::InlA^m^ with integrated pAD_1_-cGFP	(Moran et al., 2023)
*L. monocytogenes* EGDe::InlA^m^ dsRed (Lm-dsRed)	EGDe::InlA^m^ with pJEBAN6	(Moran et al., 2023)
*L. monocytogenes* EGDe::InlA^m^::*PactA*-GFP dsRed (Lm-dsRed-*PactA*-GFP)	EGDe::InlA^m^ with integrated pAD_3_-P*actA*-GFP and pJEBAN6	(Moran et al., 2023)
*L. monocytogenes* EGDe:: *actA*-ΔC dsRed *(*Lm*-actA*-ΔC-dsRed)	EGDe::InlA^m^::Δ*actA*-ΔC with pJEBAN6	This study
*L. monocytogenes* EGDe::*actA*-ΔC::*PactA*-GFP dsRed *(*Lm*-actA*-ΔC-dsRed-*PactA*-GFP)	EGDe::InlA^m^::*actA*-ΔC integrated pAD_3_-*PactA*-GFP and pJEBAN6	This study
*Listeria monocytogenes* EGDe::Δ*inlB*::GFP	EGDe::InlA^m^ Δ*inlB* with integrated pAD_1_-cGFP	This study
*Listeria monocytogenes* EGDe::Δ*actA*::*PactA*-GFP	EGDe::InlA^m^::Δ*actA with* integrated pAD_3_-*PactA*-GFP	This study

We found that during the time-course of the infection bacteria aggregate into distinct clusters (**Figures 1B**, see also **1C** and **Supplementary Movie 1**). Initially, bacteria appear to be moving freely in small clusters in the culture media, but immediately arrange into larger multi-cellular structures, which significantly increase in size over time with of up to 50 bacteria at the time of the gentamicin treatment (see **Figure 1D**). Although *L. monocytogenes* replicates in the culture media, which generally contributes to the effect, replication alone cannot explain existence of the large aggregates, since given the doubling time of 45-60 mins in rich broth (Jones and D’Orazio, 2013) approximately 2 divisions may occur before the addition of gentamicin. These aggregates not only form in the culture media but adhere to host cells and continue to increase in size after host cell binding. Simultaneously with formation of aggregates we observed upregulation of *PactA*-GFP signal, with an onset around 60 mins (**Figure 1B, C**). Bacteria appear yellow in composite images, which represents the induction of *PactA*-GFP outside host cells, in addition to constitutively expressed dsRed. During the experiment, *PactA*-GFP becomes bright green in composite images, which is especially evident at times between 2 and 6 h post infection (**Figure 1B**). This is consistent with bacteria being internalised and subsequently escaping to the cytosol, resulting in strong intracellular induction of *actA* (previous reports demonstrating 200-fold increase in transcription (Shetron-Rama et al., 2002; Chatterjee et al., 2006; Toledo-Arana et al., 2009). Overall, in agreement with the literature, our data show that prior to gentamicin treatment a subset of bacteria is able to invade host cells, where it can subsequently escape vacuole into the host cytoplasm (which can occur at the order of 10 mins (Peron-Cane et al., 2020)) resulting in rapid intracellular replication. The latter typically occurred between 2 to 4 h from the gentamicin treatment (**Figure 1B**; **Supplementary Movie 2**) and coincided with elevated *PactA*-GFP expression. Interestingly, given the high MOI, while most, if not all host cells interacted with multiple aggregates, we found that only few interactions resulted in intracellular replication of *L. monocytogenes.* Importantly, aggregation as well as ActA upregulation was also induced upon infection of primary HUVEC cells, which similarly to HeLa cells lack E-cadherin expression (Kalender et al., 2022). This demonstrates that aggregation is a general InlB-mediated phenomenon, which is physiologically relevant for human infection (**Figure 1E**).

ActA expression has been typically associated with intracellular *L. monocytogenes* (Pillich et al., 2017) and therefore the induction in *actA* transcription during aggregation suggests a role for PrfA-mediated virulence in the process. Also, aggregates of *L. monocytogenes* have been previously observed *in vitro* in culture broth and *in vivo* in the intestine during infection of mice, and have been shown to be PrfA and ActA-mediated, and specifically facilitated by the extracellular C terminal domain (amino acids-393-585) of ActA (**Figure 1F**) (Travier et al., 2013). We found that wildtype, but not *ΔprfA* nor *ΔactA* mutant strains, exhibited aggregation (**Supplementary Figure S2**). We also confirmed that the *actA-ΔC*-dsRed strain in which the C-terminus of ActA has been deleted, did not exhibit aggregation upon infection of HeLa cells (**Figures 1G, H**). Therefore, these data demonstrate that intracellular InlB-mediated invasion of *L. monocytogenes* into human non-phagocytic cells involves the formation of ActA-dependent bacterial aggregates.

### Aggregation promotes replicative invasions in HeLa and primary human cells

**Figure 2:**
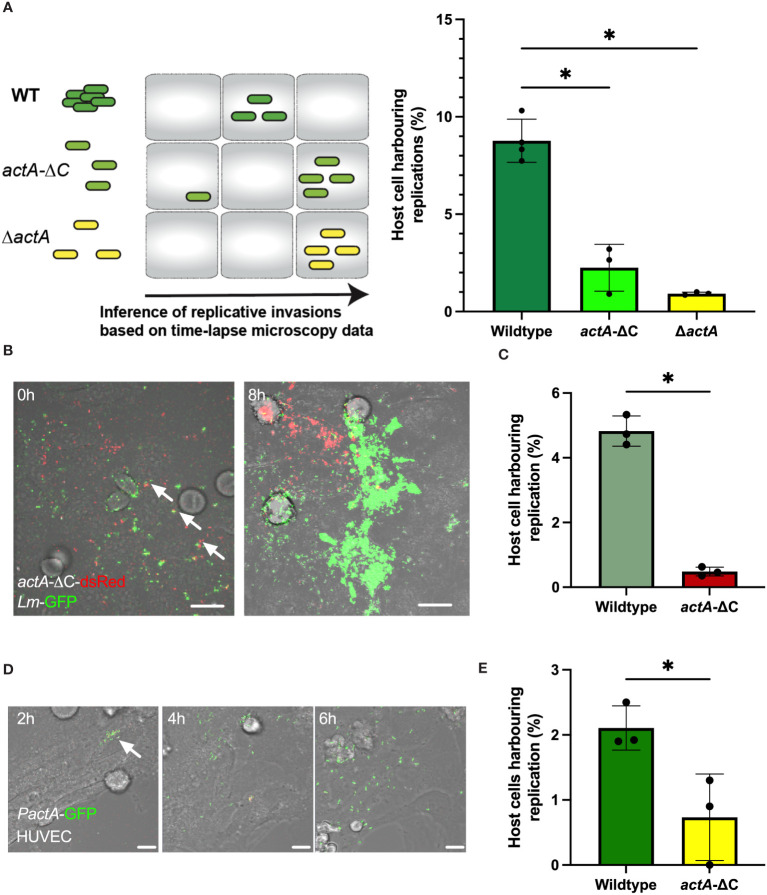
Aggregation increases probability of replicative invasion. **(A)** Percentage of HeLa cells harbouring replicative invasion events. HeLa cells infected with wildtype (*Lm*-dsRed-*PactA*-GFP), *actA-ΔC* (*actA-ΔC*-dsRed-*PactA*-GFP) and *ΔactA* (*ΔactA- PactA*-GFP) strains at MOI=20 and followed by live-cell microscopy for 8 h (see schematic diagram). Shown on the right is the fraction of host cells harbouring replicative invasion events as a function of all cells, replication inferred from time-lapse microscopy movies. Individual percentages shown in circles with mean and SD of three independent experiments as solid lines. At least 200 host cells were analysed for each replicate. Statistical significance assessed with Kruskal-Wallis test with Dunn’s correction for multiple comparisons (* = p-value ≤ 0.05). **(B)** Representative microscopy images of a co-infection experiment at indicated times. HeLa cells co-infected with wildtype *Lm*-GFP (depicted in green) and *actA-ΔC*-dsRed (depicted in red) strains at MOI=10 for each strain. Cells followed by live-cell microscopy for 8 h with gentamicin added at 2 h. Arrowheads depict aggregates composed of WT and mutant bacteria. Scale bar 10 μm. **(C)** Percentage of HeLa cells harbouring replicative invasion events from panel **(B)**. Shown is the fraction of host cells harbouring replicative invasion events as a function of all cells; replication inferred from time-lapse microscopy movies. Individual percentages shown in circles with mean and SD of three independent experiments as solid lines (with at least 100 cells per replicate). Statistical significance assessed with one-sided Mann-Whitey test (* = p-value ≤ 0.05). **(D)** Representative microscopy images of primary HUVEC cells infected with wildtype *Lm*-dsRed-*PactA*-GFP strain at MOI=5 for 2 h and assayed for further 6 h Scale bar 20 μM. Images representative of three independent experiments. **(E)** Percentage of primary HUVEC cells harbouring replicative invasion events. HUVEC cells infected with wildtype (*Lm*-dsRed-*PactA*-GFP) and *actA-ΔC* (*actA-ΔC*-dsRed-*PactA*-GFP) strains at MOI=5. Shown is the fraction of host cells harbouring a replicative invasion as a function of all cells event at 2 h after gentamicin treatment. Individual percentages shown in circles with mean and SD of three independent experiments as solid lines (with at least 100 cells per replicate). Statistical significance assessed with one-sided Mann-Whitey two-sample test (* = p-value ≤ 0.05).

We hypothesised that aggregation represents an invasion strategy. We used time-lapse microscopy movies to identify replicative invasion events by tracking host cells that were harbouring replicating bacteria, i.e., those increasing in number and robustly expressing *PactA*-GFP, confined within individual cell boundaries over the 6 h duration of the experiment. We only counted primary infection events associated with aggregate binding prior to gentamicin treatment and subsequent replication in target cell, while excluding rare and late secondary infection events associated with cell-to-cell spread or division of infected host cells. Subsequently, we compared the fraction of individual HeLa cells harbouring replicating bacteria with wildtype *Lm*-GFP to that of the non-aggregating *actA-ΔC* and *ΔactA* strains as a measure of their ability to establish replicative infections (**Figure 2A**). We found that on average 8.8% (±1.1% standard deviation) of HeLa cells facilitated intracellular replication of wildtype bacteria (calculated as a fraction of total HeLa cells) (**Figure 2A**). In contrast, only 2.3% (±1.2%), i.e., approximately 4-times less, HeLa cells were infected with the non-aggregating *actA-ΔC* strain, with 0.9% (±0.07%) cells being infected with a complete *ΔactA* mutant (**Figure 2A**).

To directly compare wild type and *actA* mutant strains, we simultaneously co-infected cells with wildtype *Lm*-GFP and *actA-ΔC*-dsRed bacteria, each at MOI=10 (see **Figure 2B** for an example of an event where both strains established replicative infections in proximity). In agreement with previous analyses, we found that the number of host cells harbouring replicative infection of the non-aggregating strain was significantly reduced in comparison to the wildtype (4.8 ±0.5% vs. 0.5 ±0.1%, respectively, **Figure 2C**). Bacteria may cooperate to manipulate host cells, for example cooperation between *Salmonella* allows otherwise non-invasive strains to enter host cells (Ginocchio et al., 1992; Kazmierczak et al., 2001; Misselwitz et al., 2012; Lorkowski et al., 2014). Although we found that some individual *actA-ΔC-dsRed* bacteria were incorporated into wildtype *Lm*-GFP aggregates (**Figure 2B**, see arrowheads), we did not find evidence that *actA*-ΔC-dsRed was able to establish replicative invasion in these aggregates. This suggests that ActA C-terminus not only facilitates aggregation but also has a role in invasion.

Finally, we wanted to determine if aggregation plays a role in invasion of primary cells. We infected primary HUVEC cells with wildtype and *actA-ΔC* reporter strains. Due to increased cytotoxicity of HUVEC cells when a large bacterial inoculum was used (Rengarajan et al., 2016), we employed a lower MOI of 5, which was sufficient to induce replicative invasions (**Figure 2D**). In agreement with assays in HeLa cells, we found that approximately 2.1% (±0.3%) of HUVEC cells harboured replicative invasions of the wildtype bacteria, whilst significantly less, 0.7% (±0.6%), harboured replicative invasions of the non-aggregating *actA-ΔC* strain (**Figure 2E**).

Overall, these data demonstrate that while replicative infections are relatively rare events (in cells negative for E-cadherin), bacterial aggregates facilitate more efficient replicative invasions compared to non-aggregating bacteria.

### Aggregates facilitate adhesion and intracellular invasion

**Figure 3:**
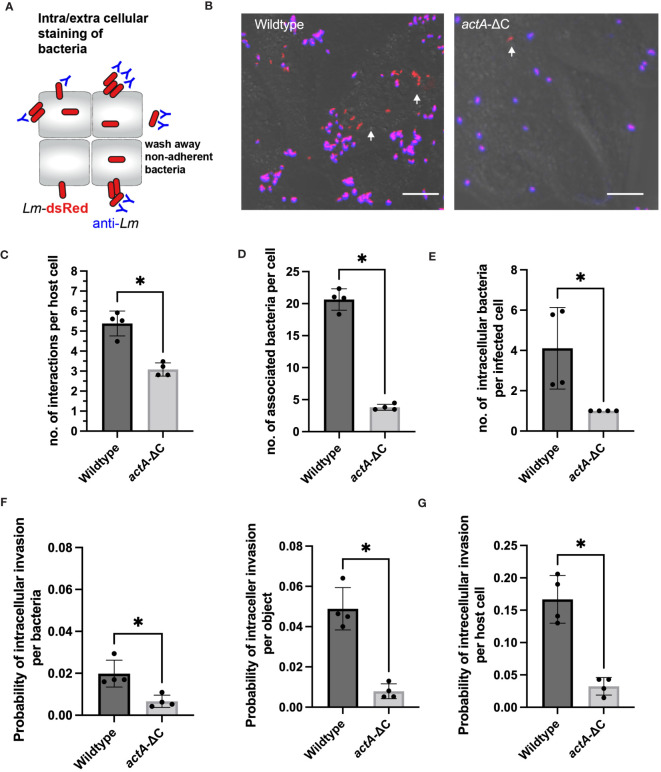
Aggregation facilitates host cell association and uptake. **(A)** Schematic representation of internalization assay using a negative anti-*Lm* antibody staining. **(B)** Microscopy images from internalisation assay of HeLa cells infected with *Lm*-dsRed (left) and *actA-ΔC*-dsRed (right) at MOI 20 and stained with anti-Lm (blue) at 2 h from *L. monocytogenes* infection. Arrowheads denote intracellular *L. monocytogenes* in red. Scale bar 5 μm. Images representative of four independent experiments. **(C)** The number of interactions per host cell (from data in B). Data represents average number of individual objects (aggregates of different size and single bacteria). Shown are individual data points (circles) across four independent experiments with solid lines indicating mean and SD. At least 100 host cells were analysed for each replicate. Statistical significance (* = p-value ≤ 0.05) assessed using Mann-Whitney two sample test. **(D)** The number of wildtype and *actA*-ΔC bacteria (from data in B) associated with host cells. Data represents the average number of bacteria for >6000 and >800 wildtype and mutant bacteria, respectively (from aggregates of different size and single bacteria) across at least 100 host cells per experiment). Shown are individual data points (circles) across four independent experiments with solid lines indicating mean and SD. Statistical significance (* = p-value ≤ 0.05) assessed using Mann-Whitney two sample test. **(E)** The number of intracellular wildtype and *actA-ΔC* bacteria (from data in B). Data represents an average number of internalised bacteria (as assessed by negative anti-*Lm* staining) in host cells harbouring intracellular bacteria. Shown are individual data points (circles) calculated for at least 100 host cells per experiment with solid lines indicating mean and SD. Statistical significance (* = p-value ≤ 0.05) assessed using Mann-Whitney two sample test. **(F)** Probability of intracellular invasion for individual wildtype and *actA-ΔC* bacteria (from data in B) calculated per individual bacteria (left) or object (right) as a function of all associated bacteria. Shown is the ratio of intracellular vs. total objects for 1517 and 659 objects in wildtype and mutant, respectively. Individual data points across four experimental replicates depicted in circles with solid lines indicating mean and SD. Statistical significance (* = p-value ≤ 0.05) assessed using Mann-Whitney two sample test. **(G)** Percentage of host cells harbouring intracellular wildtype and *actA-ΔC* bacteria from data in **(B)**. Data represents the ratio of the total host cell number with at least 1 internalised bacterium and the total number of host cells. Shown are individual data points (circles) across four independent experiments with solid lines indicating mean and SD. Statistical significance (* = p-value ≤ 0.05) assessed using Mann-Whitney two sample test.

To understand how aggregates facilitate robust intracellular replication of *L. monocytogenes*, we first characterised adherence and intracellular invasion. We used anti-*Lm* antibody staining in cells that were washed prior to the fixation protocol thus removing bacteria not associated with host cells (**Figure 3A**). This allowed distinguishing bacteria that were intracellular at 2 h post-infection from those that were adhering to the cell surface (having washed away bacteria that were not bound to host cells). In agreement with live-cell imaging (**Figure 1**), wildtype *Lm*-dsRed, but not *actA-ΔC*-dsRed strain, exhibited aggregation upon infection of HeLa cells in those assays (**Figure 3B**; **Supplementary Figure S3A**). On average, each host cell had multiple spatially resolved interactions with adhering bacteria (see the correlation between aggregate area and number of bacteria, **Supplementary Figure S3B**), but we found significantly more interactions for wildtype (5.4 ±0.6) than the mutant bacteria (3.1 ±0.3), calculated as a function of all host cells and aggregates (**Figure 3C**). In terms of actual adherent bacteria per host cell, each host cell was bound by significantly more individual wildtype *Lm*-dsRed bacteria (20.7 ±1.7) than for the *actA-ΔC*-dsRed strain (3.8±0.5) (**Figure 3D**). This demonstrates that in terms of absolute numbers, aggregated wildtype bacteria produce approximately 5-fold more host cell adhesions than the non-aggregating *actA-ΔC* mutants.

Next, we used the extracellular anti-*Lm* antibody staining to quantitatively distinguish between adherence (positive staining) and intracellular invasion (lack of staining). We found that whilst most of the bacteria were only adhering to host cells, there was clear evidence of intracellular invasion for both strains as indicated by red fluorescent signal (**Figure 3B**). These data demonstrate that for the wildtype, 70% of individual bacteria that were internalised at 2 h post-infection originated from aggregates where whole or part of an aggregate was intracellular (**Supplementary Figure S3B**). In terms of absolute numbers, we found on average 4.1 ±2 wildtype bacteria were internalised per host cell (in those host cells that were infected, i.e., harboured intracellular bacteria). In contrast, in the case of less frequent infections with *actA-ΔC*-dsRed where only one bacterium was internalised (**Figure 3E**). Based on negative and positive anti-*Lm* staining, we then counted the number of intracellular and total number of associated bacteria across recoded images to account for the different level of adherence observed in the wildtype and *actA-ΔC* strains. We found that on average, a wildtype bacterium, which was associated with a host cell, had a 0.02 ±0.006 probability of establishing an intracellular invasion (**Figure 3F**). This was approximately 3-fold higher than the corresponding probability for the *actA-ΔC* bacterium (0.007 ±0.003). Given that multiple wildtype bacteria invade from a single aggregate, probability of an intracellular invasion per aggregate was 6-fold higher (0.05 ±0.01) than that of a non-aggregating strain. Overall, this shows that aggregation not only promotes more efficient interactions with host cells, but also more efficient intracellular invasion per bacterium.

Overall, in our internalisation experiments, a host cell had 0.17 ±0.04 probability of harbouring (at least 1) intracellular invasion event, which was ~5 fold more than that of the *actA-ΔC* strain (0.03 ±0.01, **Figure 3G**). We wanted to understand if aggregation had a direct effect on intracellular replication or rather simply increased the chance of adherence and intracellular invasion. Given that we already estimated the overall probability of host cell harbouring replicative invasion (**Figure 2A**), we applied conditional probabilities to quantify if internalised wildtype and *actA-ΔC* bacteria had different abilities to replicate in the host. Using this approach, the conditional probability of harbouring a replicative invasion given that the bacteria are internalised (probability of replication of intracellular bacteria) P(R/I)=P(R)/P(I) is defined by the ratio of the overall replication event probability P(R) and the probability of internalisation P(I) (Moran et al., 2023). We found that the probability of a host cell harbouring replicative infection of intracellular wildtype *L. monocytogenes* was 0.53 ±0.14, while the equivalent probability for the *actA-ΔC* strain was 0.66 + 0.4 (based on error propagation). These numbers cannot be distinguished statistically, which suggest, that aggregation does not affect the ability of cells to replicate following invasion but that does lead to greater invasion.

### Aggregation promotes permissive InlB-MET interactions

**Figure 4:**
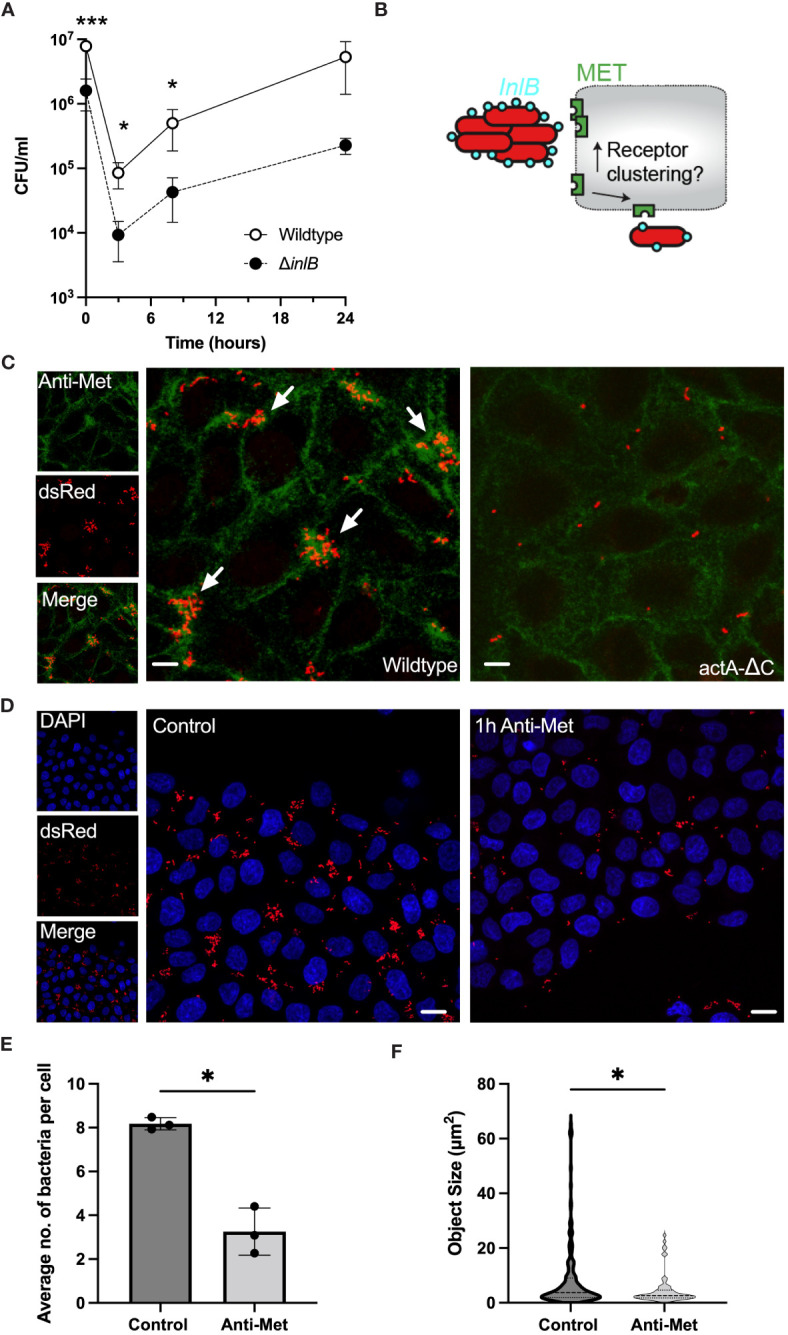
Aggregation induces robust MET interactions and clustering. **(A)** Viable bacterial counts of wildtype and *ΔinlB* strains in the gentamicin protection assays of HeLa cells (MOI =20). Bacterial counts (CFU/ml) measured at 0, 3, 8 and 24 h from addition of gentamicin, 2h after infection. The data represents means and SDs of three independent experiments. Significance assessed at 2, 5 and 10 h with one-sided t-test (* = p-value ≤ 0.05, *** = p-value ≤ 0.001). Normality assessed using Shapiro-Wilk test. **(B)** Schematic representation of the hypothesis: Does *L. monocytogenes* aggregation induce more efficient MET interaction and clustering? **(C)** Microscopy images of HeLa cells infected with *Lm*-dsRed (left) and *actA*-ΔC-dsRed (right) at MOI 20 and stained with anti-MET (green) at 2 h from *L. monocytogenes* infection. Arrowheads denote *L. monocytogenes* aggregates in red. Images representative of three independent experiments. Scale bar 5 μm. On the left are individual (red and green) channels as well as composite channel. **(D)** Microscopy images of HeLa cells infected with *Lm*-dsRed at MOI 20 either untreated (left) or pre-treated with anti-MET for 1 h before infection. DAPI staining at 2 h from *L. monocytogenes* infection shown in blue. Images representative of three independent experiments. Scale bar 10 μm. **(E)** The number of bacteria per host cell is affected by MET pre-treatment (from C). Data represents the average number of individual bacteria (a least 100 per replicate) as a function of all host cells. Shown are individual data points (circles) across replicates with solid lines indicating mean and SD. Statistical significance (* = p-value ≤ 0.05) assessed using Mann-Whitney two sample test. **(F)** Aggregate size is affected by MET pre-treatment (from C). Shown are estimated aggregate sizes (in pixels) with mean and SD based at least 180 (control) and 63 (MET pre-treatment) objects from three replicated experiments. The size distribution is represented by the grey area with interquartile range shown with dotted lines. Statistical significance (* = p-value ≤ 0.05) assessed using Mann-Whitney two-sample test.

In HeLa cells the internalisation pathway occurs mostly through the InlB-MET clathrin-mediated uptake pathway (Vessey et al., 1995; Braun et al., 1998). To validate the degree to which InlB is required in cell invasion we used a gentamicin protection assay. HeLa cells were infected with wildtype *L. monocytogenes* and the *ΔinlB* deletion mutant and after 2 hours gentamicin was added to kill extracellular bacteria. We found a significant reduction in CFU per well in the mutant bacteria, comparing to the wild type, which was maintained from addition of gentamicin for at least 8 h, consistent with the InlB-MET-mediated internalisation (**Figure 4A**). For example, at 3 hours the total number of CFU per well in the wildtype was 8.52 (± 3.71) x 10^4^ CFU/mL. In the *ΔinlB* mutant there was a significant 9-fold decrease in viable intracellular bacteria to 9.29 (± 5.74) x 10^3^ CFU/ml. This residual effect is potentially associated with previously described EDGe: InlA^m^ tropism for N-cadherin expressed by HeLa cells (Tsai et al., 2013). Overall, these analyses confirmed the invasion is predominantly occurring via the InlB-MET pathway.

Previous data suggest that InlB acts as a ‘‘molecular clamp’’ that forces the otherwise flexible MET protein into a rigid conformation through receptor clustering events, a key step leading to MET activation (Jonquières et al., 1999; Banerjee et al., 2004; Niemann et al., 2007). However, it is unclear, whether aggregates behave quantitatively different than individual bacteria, i.e., perhaps by engaging more receptors thus induce more clustering and uptake. Therefore, to further investigate the interactions between aggregates and MET we used immunostaining to measure and manipulate the cell-surface MET expression in HeLa cells upon infection with wildtype *Lm*-dsRed and *actA-ΔC*-dsRed strains (**Figure 4B**). As previously, cells were washed during the fixation protocol thus removing bacteria that were not associated with host cells. We found that adherence of wildtype aggregates was correlated with areas where MET localisation was more abundant than that of single non-aggregating *actA-ΔC* bacteria and levels of MET (**Figure 4C**; **Supplementary Figure S4A**). This suggests that bacterial aggregates promote a formation of dense MET clusters on the cell membrane, which are not present following a single bacterium interaction. To further investigate this interaction, we used a pulse and chase method to deplete the cells of available MET receptors. We hypothesised that by reducing or depleting the available MET on the cell surface we would see a reduction in the ability of wildtype *L. monocytogenes* to associate with cells. After treating HeLa cells with anti-MET for 1 hour we saw an almost total reduction in the level of MET on the cell surface (**Supplementary Figure S4B**), in agreement with a previous study (Li et al., 2019). Infection of MET-depleted cells with wildtype *L. monocytogenes* resulted in a reduction in the number and size of aggregates associated with host cells after 2 h infection, compared to control cells in which MET had not been depleted (**Figure 4D**). In these experiments, cells were also washed to remove non-adherent bacteria. Quantification of these images showed that there was a significant reduction in the average number of bacteria associated per host cell in the MET-depleted cells, from 8.17 (± 0.27) in the untreated control cells to 3.25 (± 1.07) in the MET-depleted cells (**Figure 4E**). The average size of aggregates was also reduced, from 12.6 (± 17.6) µm^2^ to 7.1 (± 8.4), equivalent to the reduction in the median from 3.7 to 2.6 and range from 64.7 to 24.4, respectively (**Figure 4F**). Overall, these data demonstrate that binding of *L. monocytogenes* is MET-dependent, while aggregates, but not individual bacteria, facilitate MET clustering resulting in more robust host cell invasion.

### Aggregation is an adaptive response to extracellular host cell environment

**Figure 5:**
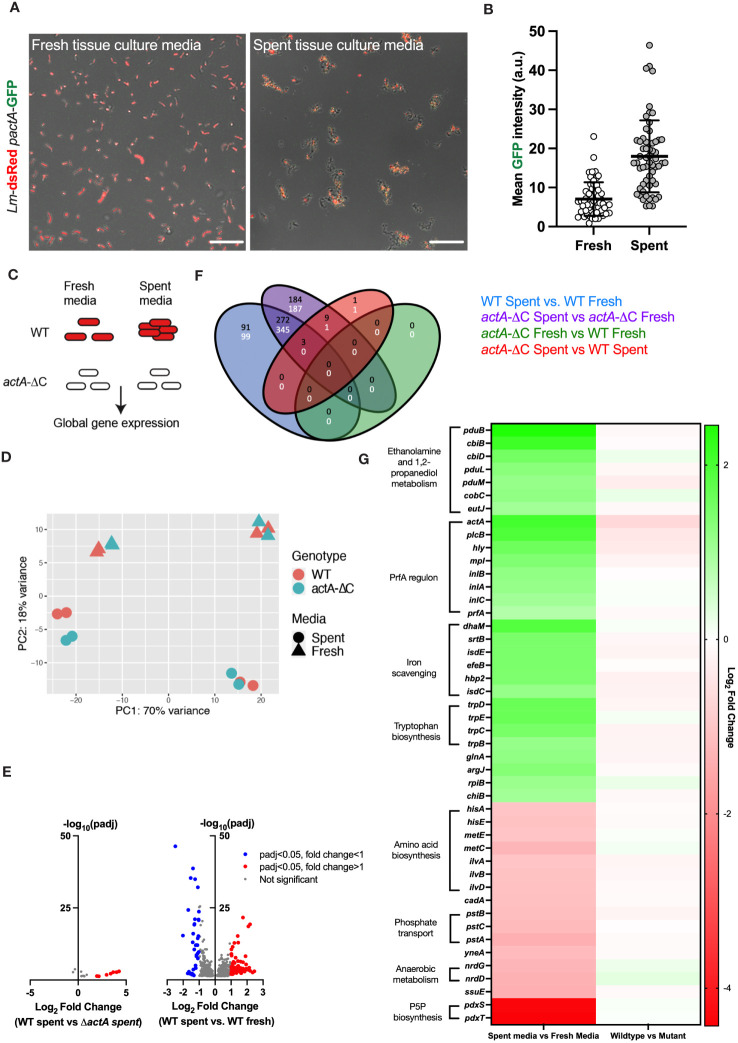
Aggregation involves a global adaptive response to host cell environment. **(A)** Representative confocal microscopy images of 1.0 x 10^7^ CFU of Lm*-*dsRed*-*P*actA-*GFP incubated in fresh or spent media (retrieved from HeLa cell) for 2 h. Shown are composite green and red channels, data representative of three independent experiments. Scale bar 10 μm. **(B)** Mean intensity of *PactA*-GFP across 60 individual objects (aggregates and individual bacteria) for each fresh and spent media from **(A)** Each dot represents an individual object, with mean and standard deviation of 3 biological replicates. Statistical analysis was performed using a Mann-Whitney test (**** = p < 0.0001). **(C)** Schematic diagram of the experiment; wild-type and non-aggregating *actA-ΔC L. monocytogenes* treated with fresh or spent media for 2 h and subjected for RNA-seq analyses. Data includes four biological replicates assayed in two batches. **(D)** Principal component analysis of the RNA-seq data from **(C)**. Shown is the relationship between genotype (WT, *actA-ΔC*) and media (spent, fresh) across 16 samples in the 1st vs 2^nd^ PCA components. **(E)** Volcano plots showing differentially regulated genes; (left) WT *L. monocytogenes* in spent media compared to *actA-ΔC* in spent media; (right) WT in spent media compared to WT in fresh media, each across 4 biological replicates. Individual genes represented in circles, thresholds set for filtering differentially expressed genes (p-adj < 0.05 and a log_2_ fold change of expression <-1 or >1) in dotted lines. Red circles represent differentially upregulated genes and blue circles represent differentially downregulated genes. **(F)** Venn diagram shows differentially regulated genes across genotype (WT and *actA*-ΔC) and type of media (fresh and spent). In black upregulated, and in white downregulated genes across different comparisons, colour coded as in the legend. **(G)** Heat map of differentially regulated genes in WT *L. monocytogenes* in spent media compared to fresh media. Shown are mean log_2_ fold-changes of read counts across 4 replicates for differentially regulated genes from **(E)**. Genes are sorted in descending order of the log2 fold changes across groups of shared ontology (as indicated by the labels). .

Our microscopy data show that *L. monocytogenes* aggregation occurs within the first 2 hours of exposure to host cells (**Figure 1C**); therefore, we hypothesised that bacteria are responding to secreted factors produced by host cells. To test this hypothesis wildtype *Lm-dsRed-*P*actA-*GFP was incubated for 2 hours in spent tissue culture media collected from HeLa cells and compared against bacteria incubated in fresh tissue culture media (see Materials and Methods for the generation of spent tissue culture media). We found that bacteria incubated in spent tissue culture media exhibited aggregation as well as upregulation of virulence gene expression, observed by GFP fluorescence from the PrfA-regulated P*actA* promoter (**Figures 5A, B**; **Supplementary Movie 3**). In contrast, when the bacteria were exposed to fresh tissue culture media, there was no aggregation nor upregulation of virulence reporter expression (**Supplementary Movie 4**).

A major advantage of aggregation being induced by spent tissue culture media was that it allowed investigations without the presence of host cells. We therefore used this system to uncover global gene expression changes in *L. monocytogenes* in response to spent and fresh tissue culture media, in the wildtype and non-aggregating *actA-ΔC* strain. We performed RNA-seq analyses of 16 samples across 4 different conditions (media vs. genotype) (**Figure 5C**). Principal component analysis (PCA) showed a robust separation between type of media (spent or fresh), but not between wildtype and non-aggregating *actA*-ΔC strain (**Figure 5D**). In addition, we observed a batch effect associated with data being collected and sequenced in two independent experiments (8 samples including two biological replicates each). A formal differential gene expression analysis (Love et al., 2014) showed 366 genes that were upregulated and 444 genes downregulated in the wildtype in spent media (compared to the fresh media control, **Figures 5E, F**, see also **Supplementary Data Dheet 1** for gene lists and analyses). In contrast, there were only 14 genes that were differentially regulated between wildtype and the *actA-ΔC* mutant strain in spent media, and none in fresh tissue culture media. This strongly suggests that spent media robustly activates gene transcription in *L. monocytogenes*, regardless of its ability to aggregate.

In terms of gene expression patterns, we found that spent media significantly induced transcription of the PrfA regulon including *actA*, *inlA*, *inlB*, *inlC*, *plcB*, *hly*, *mpl* as well as *prfA* (**Figure 5G**). For example, consistent with the imaging data, *actA* mRNA levels in the wildtype exhibited an average 4.2-fold increase in spent media, comparing to fresh media, while *plcB* and *hly* mRNA levels, required for efficient invasion (Grundling et al., 2003; Cossart, 2011) exhibited 3.8 and 3-fold increases, respectively. Both *inlA* and *inlB* exhibited statistically significant fold changes > 2.2, and while also regulated by SigB in the intestine (Toledo-Arana et al., 2009). No upregulation was observed of *sigB* regulon or other virulence genes involved in intracellular growth such as *codY*.

In addition to the PrfA regulon, we found several other systems (**Figure 5G**), which were upregulated in response to spent tissue culture media. The greatest fold increase was seen with the genes involved in ethanolamine and 1,2-propanediol metabolism (*pduB*, *pduL*, *pduM*, *cbiB*, *cbiD*, *cobC*). Genes (*srtB*, *isdC*, *isdE*, *fepB, hbp2)* encoding proteins involved in iron scavenging were also upregulated but to a lesser extent than the PrfA regulon (**Figure 5G**) as were the tryptophan biosynthesis genes (*trpB*, *trpC*, *trpD* and *trpE*), previously shown to be involved in host cell invasion (Mraheil et al., 2011). Ethanolamine and 1,2-propanediol metabolism genes were previously associated with persistent *L. monocytogenes* strains (Fox et al., 2011). Their expression was suggested to provide competitive advantage over commensal bacteria in the host due to utilisation of ethanolamine as a sole carbon source (Srikumar and Fuchs, 2011) and enhanced anaerobic growth (Zeng et al., 2019; Zeng et al., 2021). The *srtB* gene encodes Sortase B (SrtB), a second class of sortase in *L. monocytogenes*, involved in the attachment of a subset of proteins to the cell wall by recognising an NXZTN sorting motif (Bierne et al., 2004). The *isdC* and *isdE* genes present in the *svpA-srtB* operon encode for proteins that may be involved in a high affinity haem uptake system required for scavenging and Fe^2+^/Fe^3+^ binding, although the published data is somewhat conflicting regarding their function (Bierne et al., 2004). The *fepB* gene encodes for deferrochelatase, which is involved in the recovery of exogenous haem iron while *hbp2* gene encodes for a protein involved in haem acquisition (Lechowicz and Krawczyk-Balska, 2015).

Significantly downregulated genes in spent tissue culture media included genes with roles in amino acid (*hisA*, *hisE*, *metC*, *metE*, *ilvA, ilvB, ilvD*) and pyridoxal 5’- phosphate P5P (*pdxS*, *pdxT*) biosynthesis, phosphate transport (*pstA*, *pstB*, *pstC*) and anaerobic metabolism (*nrdG*, *nrdD*) (**Figure 5G**). P5P is an essential cofactor for numerous metabolic enzymes (Belitsky, 2004). The *pstA, pstB* and *pstC* genes encode transmembrane proteins involved in the phosphate transport system (Moreno-Letelier et al., 2011; Choi et al., 2015). Catalytic subunit *nrdD* and activase *nrdG* encode class III anaerobic ribonucleotide reductase used in anaerobic conditions, however this NrdD protein has impaired function in EGD-e due to a 6 amino acid deletion (Ofer et al., 2011).

Taken as a whole, these data indicate responses by *L. monocytogenes* to likely changes in available nutrients present in the spent tissue culture media. In particular, the observed activation of iron uptake systems suggests that bacteria are adapting to changes in iron concentration in the media. By inductively coupled plasma mass spectrometry (ICP-MS) we determined that the iron concentration in spent media was 0.2 (± 0.007) μM, which was significantly lower than in the fresh media (0.39 ± 0.007 μM, **Supplementary Figure S5**). Given the well-known effect of iron depletion on upregulation of PrfA activity (Conte et al., 2000; McLaughlin et al., 2011), this suggests that depletion of iron levels in the spent media may contribute to PrfA-mediated aggregate formation.

Overall, these data demonstrate the aggregation is an adaptive response to factors produced by host cells, resulting in metabolic changes in *L. monocytogenes* and upregulation of PrfA virulence, which drives aggregation and facilitates intracellular invasion.

## Discussion

In this work we used live-cell microscopy approaches to investigate the InlB-mediated invasion strategy of *L. monocytogenes* of human non phagocytic cells. We found that replicative invasion of *L. monocytogenes* in HeLa and human primary HUVEC cells are generally very rare events, where in conditions of high MOI (average of 20 bacteria per host cell) only <1% bacteria and only <10% of host cells harbour replicative invasions. Although our findings involve InlB-mediated host cell entry (using cells that do not express E-cadherin), this low rate of invasion for *L. monocytogenes* is reflected by other studies where high MOIs are required to infect non phagocytic cells *in vitro* (Velge et al., 1994; Chico-Calero et al., 2002; Grundling et al., 2003; Pentecost et al., 2006; Wang et al., 2015; Yin et al., 2019; Rengarajan and Theriot, 2020; Abdulkadieva et al., 2023). Whether E-cadherin-mediated host cell entry also relies on aggregation was not investigated in this manuscript and remains to be seen. In general, probabilistic outcomes of the single cell host pathogen interactions may be the consequence of heterogeneity of host and pathogen at the single cell level, involving stochastic gene expression or signalling events in the host or pathogen or both, which together control infection outcomes (Avraham et al., 2015; Guldimann et al., 2017; Bagnall et al., 2020; Rengarajan and Theriot, 2020; Moran et al., 2023). Here we demonstrate that *L. monocytogenes* overcomes these low invasion rates by employing a simple, yet effective strategy relying on extracellular aggregation, enabling more robust adhesion and host cell entry. We specifically show that compared to wildtype, the non-aggregating *actA-ΔC* mutant bacteria was impaired in its ability to form replicative invasions in HeLa cells (4-fold reduction) and primary HUVEC cells (3-fold reduction) and was outcompeted by the wildtype in a co-infection assay. Aggregation as an invasion strategy has been previously described for *Bartonella henselae* (Dehio et al., 1997) and *Pseudomonas aeruginosa* (Lepanto et al., 2011). ActA-dependent aggregation has been also shown for *L. monocytogenes in vitro* (in bacterial growth medium) as well as *in vivo*, in the gut lumen upon oral infection of mice where it promoted long-term bacterial persistence within gut lumen (Travier et al., 2013). Therefore, to our knowledge this is the first report that describes the role of ActA-mediated aggregation in *L. monocytogenes* invasion. ActA is involved in crossing fetoplacental (Le Monnier et al., 2007) and the blood-brain barrier (Ireton et al., 2021), internalisation into human mononuclear trophoblasts (Johnson et al., 2021) as well as a range of epithelial cell lines (Suárez et al., 2001), however this is usually shown or assumed via its role in cell-to-cell spread. Here we propose that these ActA-mediated mechanisms, involving InlB interactions (Dramsi et al., 1995) might at least in part be a consequence of aggregation at different sites outside of the intestine, where the MOI of bacteria might rapidly increase in a local tissue environment.

Why most of the interactions between *L. monocytogenes* and host cells do not result in replicative invasion and what are the characteristics of successful interactions leading to intracellular invasion are not fully known. Ultimately, the rate of invasion, might depend on the state of individual host cells and reflect host tissue differences, e.g., with respect to MET/Ecad expression or signalling (Bhalla et al., 2019). In turn, robust expression of PrfA-mediated InlB/A by bacteria is likely required to facilitate a functional interface with the host receptors (Niemann et al., 2007; Abdulkadieva et al., 2023). Previous analyses suggest that PrfA (and presumably InlB/A) activation is heterogenous (Guldimann et al., 2017; Moran et al., 2023), which might be a limiting factor for permissive host-cell interactions. Our data show that *L. monocytogenes* aggregates induce 5-fold more host cell interactions than the non-aggregating *actA-ΔC* strain. For those bacteria that are associated with host cells, aggregates induced 3-fold more intracellular invasions than the non-aggregating strain ultimately leading to increased rate of replicative invasion. In part, this is mediated by the ability of aggregates to support multiple invasion events into a single host cell. We must note that our calculations (**Figure 3**) do not take into account the distribution of aggregate sizes, since establishing associations between specific extracellular aggregates and intracellular bacteria would not be robust based on fixed cell imaging data. Even in the WT *L. monocytogenes* infection 30% internalisation seems to occur from the aggregate of size 1 (**Supplementary Figures S3A, B**), we likely underestimate the effect of aggregation facilitating invasion for those bacteria associated with host cells. We showed that aggregates induce receptor clustering, thus potentially producing more stable interactions with receptors, comparing to non-aggregating mutant. Whether this is related to increased local InlB concentration at the receptor complex remains unknown. Our data clearly indicate that ActA and InlB are critical for host cell entry, while likely the elevated levels due to PrfA activation facilitate better invasion. While aggregates may form without a physical presence of host cells (**Figure 5A**), MET is required for host cell biding (**Figure 4D**). We therefore hypothesise that *L. monocytogenes* aggregates enable efficient engagement of multiple MET receptor dimers, resulting in more robust entry. These could be achieved by receptors efficiently “scanning” through multiple bacteria “presented” via the aggregate, mimicking to some extent the immunological synapse (Dustin, 2014). On the pathogen side, aggregation might allow localised increases in otherwise heterogenous PrfA activity (Guldimann et al., 2017) thereby increasing effective internalin concentration at the interface between *L. monocytogenes* and host receptor systems.

Biofilm formation and presumably aggregation may play important roles in the pathogenesis of human diseases (Vestby et al., 2020) as well as the food industry by contributing to food contamination (Carpentier and Cerf, 2011). Previously, different virulence factors including the PrfA regulon (Lemon et al., 2010; Zhou et al., 2011), the stress activated SigB regulon (van der Veen and Abee, 2010) as well as quorum sensing LuxS and Agr systems (Challan Belval et al., 2006; Rieu et al., 2007) have been implicated in *L. monocytogenes* biofilm formation. We used our spent host cell media experiments to investigate changes in *L. monocytogenes* upon exposure to the host cell environment, thus extending previous analyses typically relaying on intracellular bacteria or response to bacterial culture media (Toledo-Arana et al., 2009; Reniere et al., 2015). Our live-cell analyses demonstrate that upon exposure to spent host cell media *L. monocytogenes* induces transcription of several genes in the PrfA regulon, including *actA* and *inlB* required for aggregation and internalisation. This extends previous analyses demonstrating *ActA*-mediated *L. monocytogenes* aggregation in bacterial media (Travier et al., 2013), but also specifically demonstrates that aggregation in the context of infection is a response (within the 2 h from infection) to the host cell environment. This may be a consequence of factors secreted rapidly by the host cells and/or changes in the culture media as a result of host cell metabolism. We considered a possibility that aggregation plays a role in upregulation of PrfA virulence, but the non-aggregating *actA-ΔC* strain exhibited transcriptional profiles similar to that of the wildtype cells, suggesting a general response to host cell environment. This suggests a model where factors in the extracellular milieu outside host cells drives expression of PrfA regulon including ActA, thus enabling aggregation and subsequently a more robust host cell invasion, in comparison to non-aggregating strains. Based on the induction of genes involved in iron acquisition we predicted and demonstrated that spent tissue culture media is low in iron (in comparison to fresh media). It has been shown that expression of the PrfA regulon is induced when iron is depleted in the culture media (Gaballa et al., 2021), which provides a potential explanation for the increased expression of the PrfA regulon and the induction of ActA-mediated aggregation. It has been also shown that induction of the *trp* operon occurs in *L. monocytogenes* during growth in interferon gamma (IFNγ) treated macrophages, likely in response to low levels of intracellular tryptophan through increased activation of the kynurenine pathway in IFNγ treated macrophages (Mraheil et al., 2011). In addition, the observation that the presence of exogeneous tryptophan in *L. monocytogenes* growth media decreased expression of the *trp* operon (Mraheil et al., 2011) confirms that the levels of tryptophan play a role in the regulation of *trp* gene expression and would indicate spent culture media is low in tryptophan.

In summary our data suggest a mechanism whereby the extracellular host environment primes the bacteria to switch to an intracellular gene expression state, promoting the activation of the PrfA regulon and ActA, which subsequently facilitates aggregation resulting in more robust InlB-dependent invasion of host cells.

## Materials and methods

### Bacterial strains

We used *L. monocytogenes* EDGe: InlA^m^ (Wollert et al., 2007), suitable for animal studies; this mutation is not affecting the binding with MET investigated herein. Wildtype and mutant *L. monocytogenes* EDGe: InlA^m^ were grown at 37°C shaking (200 rpm) in tryptone soya broth (TSB) (Oxoid) or with additional 1.5% (w/v) agar (Oxoid) unless otherwise stated. *L. monocytogenes* mid-log (OD_600_ 0.5-0.6) aliquots stored at -80 °C in PBS glycerol (15% v/v) were used for infections. *Escherichia coli* DH5α grown in Luria-Bertani broth (LB) was used for cloning and when required media was supplemented with antibiotic (Erythromycin 5 μg/ml, Chloramphenicol 7 μg/ml).

Plasmids were electroporated into *L. monocytogenes* to generate fluorescently tagged and fluorescent reporter strains (see **Tables 1** and **2** for plasmids and strains used in the study). Chromosomal integration of integrative plasmids at the tRNA^Arg^-*attBB* site was confirmed by PCR as described previously (Lauer et al., 2002). Correct fluorescence of strains was confirmed by microscopy. *L. monocytogenes* Δ*actA* and *actA*-ΔC mutant strains were constructed using the temperature sensitive shuttle plasmid pAUL-A as described previously (Wang et al., 2015). Viability of stored bacteria was confirmed by routine measurements of colony forming units of the inoculum.

### Cell Culture

HeLa cells were grown in Dulbecco’s modified eagle media (DMEM, Sigma) supplemented with 10% (v/v) foetal calf serum (FCS, Gibco) and 1% (v/v) non- essential amino acids (Gibco) at 37°C 5% CO_2_ (v/v). Cells were maintained by sub- cultivating at a ratio between 1:2 to 1:6 split 2-3 times a week depending on cell density. HUVEC cells (Thermo Fisher) were recovered from cryopreservation and grown in Human Large Vessel Endothelial Cell Basal Medium (HLVEM, Thermo Fisher) supplemented with Large Vessel Endothelial Supplement (Thermo Fisher) at 37°C 5% CO_2_ (v/v) for no more than 16 doublings after the initial recovery, as per the provider recommendations. Infections are performed in serum-free media to avoid opsonisation of bacteria preventing host cell binding.

### Gentamicin protection assay

HeLa cells were seeded in DMEM supplemented with 10% FCS at a density of 2.0 x 10^5^ in a 6 well plate and incubated overnight at 37°C 5% CO_2_ (v/v). Subsequently media was aspirated, and the cells were washed in PBS. Serum-free DMEM containing 1.0 x 10^7^ CFU/ml *L. monocytogenes* was added at a MOI of 20. The plates were incubated for 2 hours at 37°C 5% CO_2_ (v/v). After washing in 2 ml of PBS twice, 2 ml of serum-free DMEM was added except the wells being analysed for the first time point. Remaining plates were then incubated with gentamicin (at 10 μg/ml final concentration) until the required time point. Bacteria were retrieved and enumerated by washing the wells in 1 ml of PBS three times and then adding 1 ml of PBS with 0.5% (v/v) triton to lyse the host cells for 2 mins. This solution was then mixed and transferred to a 1.5 ml Eppendorf tube. A serial dilution up to a dilution factor of 10^-5^ was performed in PBS and 20 μl of all the dilutions was transferred in triplicate to a TSB agar plate. Plates were left to dry and then transferred to an incubator and incubated overnight at 37°C. Colonies were counted and multiplied by the dilution factor to retrieve the CFU/ml for each well.

### Live-cell microscopy infection assays

Cells were seeded in media (DMEM for HeLa and HLVNM for HUVEC) supplemented with 10% (v/v) FCS into a 35 mm imaging dish (Griner Bio One) at 2.0 x 10^5^ and incubated overnight. Cells were infected with *L. monocytogenes* prepared in 3 ml of pre-warmed serum-free media at MOI 20 (or 5 for HUVEC cells) for 2 h and washed three times with PBS prior to the addition of gentamicin containing serum-free media (at final concentration 10 μg/ml). Infections were imaged by live-cell time-lapse microscopy using a Zeiss LSM880 microscope using 40x (1.4NA) oil immersion objective and 488 nm laser for GFP, and 561 nm for dsRed fluorophores. Z-stack function was used for imaging, taking 4 slices across 12.24 μm between the first and last slice, every 3 mins per location. Zeiss Zen Black software was used to generate a maximum intensity projection single plane image from the Z-stacks. Replicative invasion events refer to individual host cells exhibiting increasing number of *PactA-GFP*-expressing bacteria inferred by visually tracking host cells and bacteria in the captured live-cell microscopy movies over the duration of the experiment. Initial infection events were typically spatially separated, allowing robust identification of secondary infections associated with cell-to-cell spread and division of infected host cells. Secondary infections were not considered in our analyses. In some bacteria the constitutive dsRed signal (expressed from non-integrative plasmid) may disappear due to rapid proliferation and presumably insufficient production of the fluorescent marker. Probability of replication was calculated as the number of successful replicative invasions in relation to the total number of host cells present in the image at the first time point. Aggregate size and intensity were measured by using the ROI (region of interest) manager in FIJI (Schindelin et al., 2012). Individual aggregates were segmented in the image by drawing around the edge using the freehand tool. These were recorded in the ROI manager and measured for their size or intensity, when relevant.

### Generation of spent media and aggregation assay

2.0 x 10^5^ HeLa cells were seeded into an 35mm imaging dish (Greiner Bio One) in DMEM supplemented with FCS and L-glutamine and incubated in 37°C 5% CO_2_ (v/v). The next day cells were washed with PBS and supplemented with serum-free DMEM (no FCS). The following day, 1 ml of spent media (at 37°C) from above cultures was added to imaging dish and the bacterial inoculum was added to a final cell density of 1.0 x 10^7^ CFU/ml. In some experiments a control was also performed with fresh serum free DMEM media. The dish was sealed with parafilm and either incubated at 37°C 5% CO_2_ (v/v) in a cell culture incubator or on the incubation unit of LSM 880 while imaged. A time series was taken every 15 seconds in an individual tile with brightfield, 488 nm and 561 nm lasers using the z-stack function taking 4 slices with 12.24 μM distance between the first and last slice. In non-fluorescent strains the 488 nm laser and brightfield was used for analysis. Aggregate size (in μm^2^) were measured by using the ROI manager in FIJI, individual bacteria were segmented in the image by drawing around the edge using the freehand tool. Single cell virulence expression was obtained using mean intensity of the fluorescent reporter signal per segmented object.

### Internalisation assay

HeLa cells were cultured in an imaging dish to a density of 2.0 x 10^5^ cells and incubated overnight. Cells were washed with PBS and 1 ml of the media containing bacteria was added to the imaging dish at the desired MOI and incubated at 37°C 5% CO_2_ (v/v) for 2 hours. Media was removed and the cells were washed twice with PBS. 1 ml of 4% (v/v) paraformaldehyde (PFA) suspended in PBS was added and the dishes at room temperature for 20 mins. The PFA was removed, and the cells were washed 3x in PBS. The cells were incubated with blocking buffer (1% (v/v) BSA, 22.52 mg/ml glycine, 0.1% (v/v) tween-20) for 30 mins and washed with PBS three times. 300 μl of diluted (1:500 from supplier stock - 8 ug/ml) rabbit anti-Listeria antibody (Abcam, ab35132) was added and incubated at room temperature for 20 mins. Dishes were washed 3x in PBS and then secondary antibody donkey anti-rabbit IgG Brilliant Violet 421 (Biolegend) was added and incubated at room temperature for 20 mins and then washed 3x in PBS. Images were taken with a Zeiss 880 confocal microscope with 40x (1.4 NA) immersion oil objective using appropriate lasers. Z-stack images were taken with 5 slices and 12.24 μm between the first and last slices. Images were analysed in FIJI by manually counting the number of bacteria in each aggregate or using the ROI manager to obtain aggregate size (see **Supplementary Figure S3B** for correlation between aggregate size and number of bacteria). The size (in μm^2^) of each external/internal aggregate, number of bacteria, total number of aggregates and total number of host cells were recorded. Internalised bacteria were recorded as those objects that were excited by the 561 nm laser (dsRed) but were not excited by the 405 nm laser settings, i.e., they had been internalised as not stained with the anti-Listeria antibody. The size of each external/internal aggregate, number of bacteria, total number of aggregates and total number of host cells were recorded. The number of associated bacteria per cell was calculated by taking the total number of associated bacteria and dividing the total number of host cells. Probability of invasion was calculated by dividing the number of intracellular bacteria by the total number of associated bacteria.

### MET depletion and staining of HeLa cells

HeLa cells cultured in an imaging dish to a density of 2.0 x 10^5^ were incubated overnight. Cells were washed twice with PBS and 1 ml of 4% paraformaldehyde (v/v) suspended in PBS was added and incubated at room temperature for 20 mins. The PFA was removed, and the cells were washed 3x in PBS. The cells were incubated with blocking buffer (1% (w/v) BSA, 22.52 mg/ml glycine, 0.1% (v/v) tween-20) for 30 mins and washed with PBS three times. 5 μg/ml of primary goat Anti-MET antibody (Abcam) suspended in blocking buffer was added to the dish and left for 1 min. The cells were washed 3x with PBS by adding the PBS and leaving it for 20 mins before removing and changing the PBS. After the washes the secondary antibody 10 μg/ml Alexafluor 488 anti-Goat (Abcam) was added and incubated at room temperature for 30 mins. The cells were washed 3x with PBS for 20 mins per wash. DAPI Vectashield was added to the dish to preserve fluorescence and to stain cell nuclei with DAPI. The cells were imaged using a Zeiss 880 confocal microscope and a 40x objective (1.4NA) oil immersion lens. 488 nm laser was used for excitation settings and background signal was removed by calibrating against a negative control with no staining. To deplete the cell surface of MET, 2 μg/ml of primary antibody was added to serum free DMEM and this was incubated on the cells for 1 hour at 37°C 5% CO_2_ (v/v). After MET depletion *L. monocytogenes* was added to the host cells at a MOI of 20 in serum free media and incubated for 2 hours at 37°C 5% CO_2_ (v/v) before fixing and imaging as described above. ROI manager in FIJI was used to quantify MET staining associated with bacteria, using freehand tool to segment bacteria and measure GFP intensity in the corresponding region.

### RNA extraction and preparation

RNA extraction was performed using Purelink RNA Extraction Kit (Invitrogen) and Lysing Matrix E tubes (MP Biomedicals. Fresh lysozyme solution (10 mM Tris-HCl, 0.1 mM EDTA, 10 mg/ml lysozyme), 10% SDS (v/v) in RNAse-free water and lysis buffer (supplemented with 10 μl of 2-mercaptoethanol per 1 ml of buffer) were prepared. Bacteria were pelleted by centrifuging at 13000 x *g* for 3 mins and the supernatant was discarded. 100 μl of lysozyme solution was added and the pellet was resuspended by vortex. 0.5 μl of 10% (v/v) SDS solution was added and mixed via vortex. 350 μl of lysis buffer was added and mixed by vortex. The lysate was transferred to a Lysing Matrix E tube and homogenised in a rotor stator homogeniser (FastPrep FP120) at 6.5 speed setting for 45 seconds. The tube was centrifuged at 2600 x *g* for 5 mins and the supernatant was transferred to a RNAse-free microcentrifuge tube. 250 μl of 100% (v/v) ethanol was added to the tube and mixed by vortex to remove precipitate. The sample was transferred to a kit spin cartridge and centrifuged at 12000 x *g* for 15 seconds to bind RNA to column. Flow-through was discarded and 700 μl of Wash Buffer 1 was added to the column. The column was centrifuged at 12000 x *g* for 15 sec and both the flow through and collection tube were discarded. The column was placed in a new collection tube and 500 μl of Wash Buffer 2 was added to the column. This was centrifuged at 12000 x *g* for 15 sec, and flow-through was discarded. The wash step with Wash Buffer 2 was repeated and after flow-through was discarded again the column was centrifuged at 12000 x g to remove any residual wash buffer. The column was transferred to a recovery tube (RNAse free 1.5ml tube provided by kit) and 50 μl of RNAse free water was carefully added directly to the column membrane. This was incubated for 1 min at room temperature to elute RNA and then centrifuged at 12000 x *g* for 2 mins. All downstream work with RNA after this point was performed on ice.

Genomic DNA (gDNA) was removed using TURBO DNAse kit. 0.1 volume of 10x TURBO DNAse buffer and 1 μl of TURBO DNAse was added the RNA samples and gently mixed. The samples were incubated in a 37°C water bath for 30 mins. The solution was mixed by vortex and 2 μl of DNAse inactivation reagent was added. The samples were incubated for 5 mins at room temperature and flicked 2-3 times during incubation to keep inactivation reagent suspended throughout the mixture. Samples were centrifuged at 10,000 x *g* for 90 sec and the supernatants were transferred to a fresh RNAse free collection tube. Samples were frozen at -80°C until ready for sequencing. RNA integrity, concentration, and presence of gDNA using an Agilent Tapestation System.

### RNA-seq

RNA-Seq was performed by the Genomic Technology Core Facility. A total 16 samples across 4 different conditions (media vs. genotype) were prepared in two independent experiments performed (and sequenced) at different times (8 samples each including two independent replicates). A ribosomal Stranded Total RNA Prep with Ribo-Zero Plus depletion kit (Illumina) was used with custom probes (Integrated DNA Technologies) against the *L. monocytogenes* ribosomal RNA subunits. These were combined with the standard depletion probes (DP1) at the ‘Hybridise Probes’ step of the protocol, as per manufacturer’s instructions. An RNA sample prep kit (Illumina Stranded Total RNA Prep) was used to fragment and denature RNA, synthesise first and second strands, adenylate 3’ prime ends, ligate anchors, clean up fragments and amplify and clean up the library. Additional probes to efficiently deplete the rRNA from *L. monocytogenes* were added during the probe hybridization step on recommendation from in silico analysis performed by Illumina. Libraries were added to an equimolar pool which was quantified by qPCR (KAPA). The pool was then denatured and loaded onto one lane of an SP NovaSeq 6000 flowcell using the XP workflow, at 200pM with 1% (v/v) PhiX spiked in. The NovaSeq 6000 was run in XP mode with (sequencing lengths: Read 1: 59bp, i7 index: 10bp, i5 index: 10bp, Read 2: 59bp). Unmapped paired-end sequences were tested by FastQC. Sequence adapters were removed, and reads were quality trimmed using Trimmomatic_0.39 (Bolger et al., 2014). The reads were mapped against the reference *L. monocytogenes* EGDe Genome and annotation. Counts per gene were calculated using featureCounts (subread_2.0.0) (Liao et al., 2013). Normalisation, Principal Components Analysis, and differential expression was calculated with DESeq2_1.36.0 (Love et al., 2014).

### Metal analysis

The levels of iron were determined by ICP-MS as described before (Corbett et al., 2011). 1 ml of fresh and spent media samples were diluted into 2% nitric acid and analysed in three replicate experiments.

### Statistical analyses

Statistical analysis was performed using GraphPad Prism 9 software. Data were tested for normal distribution using Shapiro-Wilk test. Two-sample comparisons were conducted using paired t-test for data that was normally distributed, otherwise non-parametric Mann Whitney test was used. Multiple comparisons were analysed using ANOVA for normally distributed data, otherwise Kruskal-Wallis ANOVA was used.

## Data availability statement

The datasets presented in this study can be found in online repositories. Generated sequencing data have been deposited in the ArrayExpress database at EMBL-EBI under accession number E-MTAB-13081 (https://www.ebi.ac.uk/biostudies/arrayexpress/studies/ E-MTAB-13081). Raw imaging data in Carl Zeiss file formats have been deposited under 10.5281/zenodo.10049916.

## Author contributions

LF: Conceptualization, Data curation, Formal analysis, Investigation, Methodology, Validation, Writing – review & editing. JM: Investigation, Methodology, Supervision, Writing – review & editing. MG: Investigation, Methodology, Writing – review & editing. EL: Investigation, Methodology, Writing – review & editing. DS: Methodology, Writing – review & editing. JC: Methodology, Supervision, Writing – review & editing. MM: Funding acquisition, Supervision, Writing – review & editing. IR: Conceptualization, Funding acquisition, Methodology, Supervision, Writing – original draft, Writing – review & editing. PP: Conceptualization, Funding acquisition, Methodology, Project administration, Supervision, Writing – original draft, Writing – review & editing.

## Supplementary material

Supplementary Data Sheet 1 Differentially regulated genes across genotype and media type.

Supplementary Data Sheet 2 Tabularized manuscript data.

Supplementary Movie 1 Formation of aggregates and upregulation of virulence genes upon host cell infection. Representative confocal microscopy video of 3 independent experiments of HeLa cells infected with Lm*-*dsRed*-PactA-*GFP MOI=20 for 2 h. Video show the formation of bacterial aggregates and upregulation of virulence gene expression as shown by increased GFP expression. Scale bar 5 μm.

Supplementary Movie 2 Replicative invasion of host cells. Representative confocal microscopy video of 3 independent experiments of HeLa cells infected with Lm*-*dsRed*-PactA-*GFP MOI=20 for 2 h. Scale bar 10 μm.

Supplementary Movie 3
*L. monocytogenes* exposed to spent host cell media. 1.0 x 107 Lm-dsRed-*PactA*-GFP was incubated for 2 h at 37°C in spent media. Representative video of 3 independent experiments show upregulation of PrfA-regulated virulence genes via *PactA*-GFP (green) expression and the formation of aggregates. Scale bar 10 μm.

Supplementary Movie 4
*L. monocytogenes* exposed to fresh media.1.0 x 107 Lm*-*dsRed*-*P*actA-*GFP was incubated for 2 h at 37°C in fresh media (bottom). Representative video of 3 independent experiments show upregulation of PrfA-regulated virulence genes via *PactA-*GFP (green) expression but no aggregation occurs when exposed to fresh media. Scale bar 20 μm.
