# Supplementary material for: Bacterial aggregation facilitates internalin-mediated invasion of Listeria monocytogenes
Source: Front Cell Infect Microbiol. 2024 Jul 9;14:1411124. doi: 10.3389/fcimb.2024.1411124 (PMC11263170; doi:10.3389/fcimb.2024.1411124)
Supplement: Supplementary file 1 [file Presentation_1.pdf]

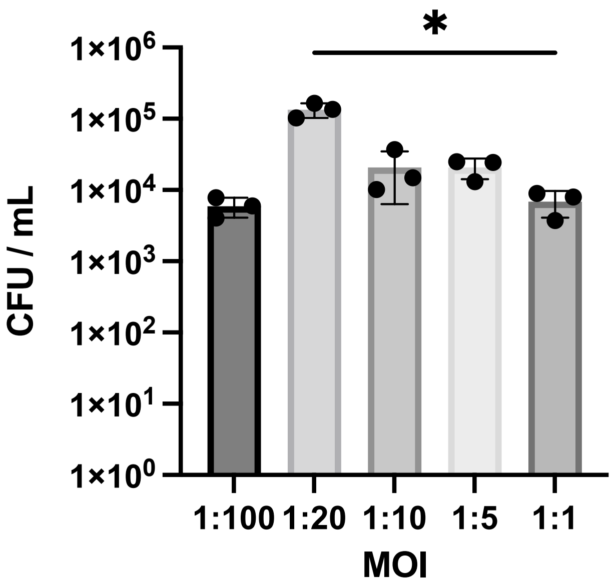

Figure S1. **Intracellular growth of *L. monocytogenes* depends on MOI.** Viable bacterial counts of wildtype *L. monocytogenes* in the gentamicin protection assays of HeLa cells across a range of MOIs (as indicated on the graph). Bacterial counts (CFU/ml) measured at 8 h from infection, with gentamicin added at 2 h. The data represents means and SDs of three replicated experiments. Statistical analysis was performed using Kruskal-Wallis test with Dunn's correction for multiple comparisons. (* = $p < 0.05$).

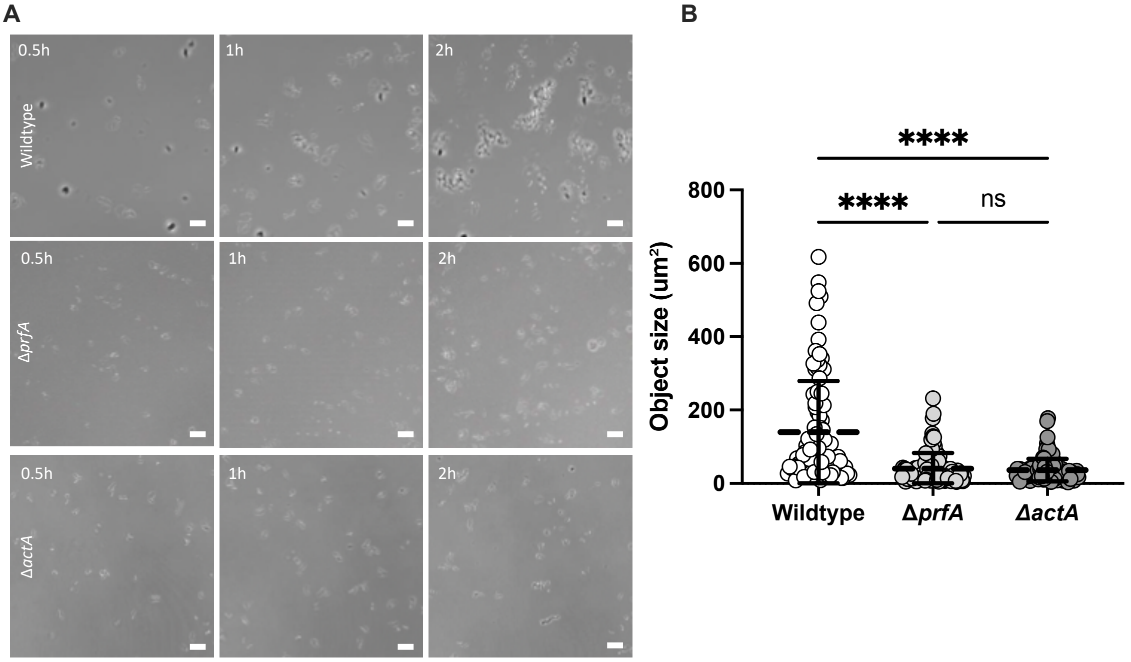

Figure S2. Aggregation requires PrfA and ActA. A) Representative images showing 1.0 x 10^7 CFU of wildtype *L. monocytogenes*, *ΔprfA* mutant and *ΔactA* mutant incubated for 2 h at 37°C in spent media from HeLa cells. Images representative of three experimental replicates. Scale bar 5 µm. **B)** Aggregate area for strains in A. Shown are individual objects in μm^2 (>100 per condition) as circles with mean and standard deviation based on three replicates. Statistical analysis was performed using Kruskal-Wallis test with Dunn's correction for multiple comparisons. (**** = $p < 0.0001$ ns = not significant).

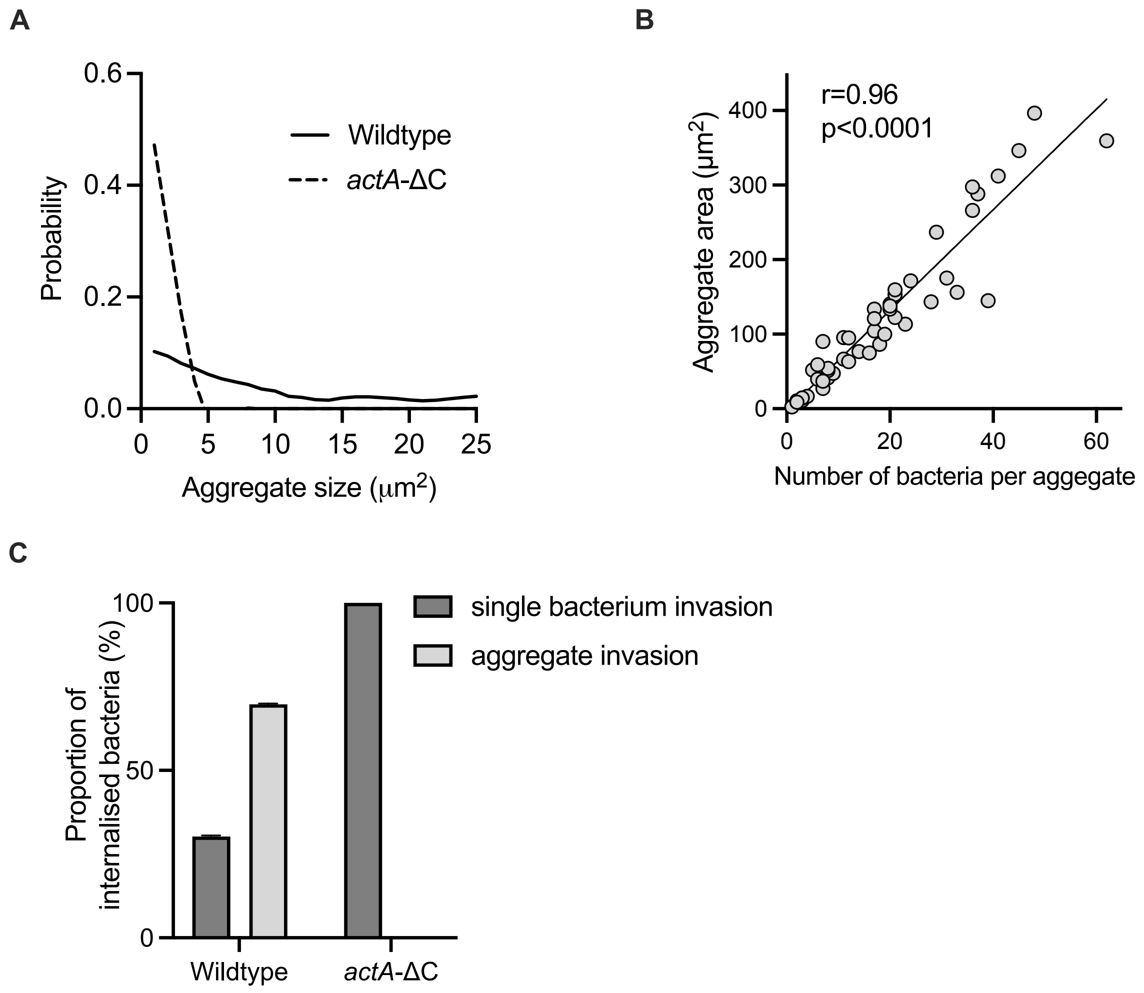

Figure S3. Microscopy analysis of wildtype and *actA-ΔC* bacteria. A) Correlation between aggregate area (in μm^2) and number of wildtype bacteria. Shown are 50 representative segmented objects from data in Fig. 3B (in circles) as well as a linear regression fit (in black). p-value depicts a result of a statistical test for a positive Spearman's correlation (r). **B**) Probability distribution of aggregate size (μm^2) for the wildtype and the non-aggregating *actA-ΔC* mutant. Data from Fig. 3A is pooled from 4 biological replicates. **B**) Analysis of intracellular bacteria from Fig. 3A categorised into either as a single cell invasion where a single bacterium has successfully invaded a host cell, or an aggregate invasion where multiple bacteria have successfully invaded the host cell.

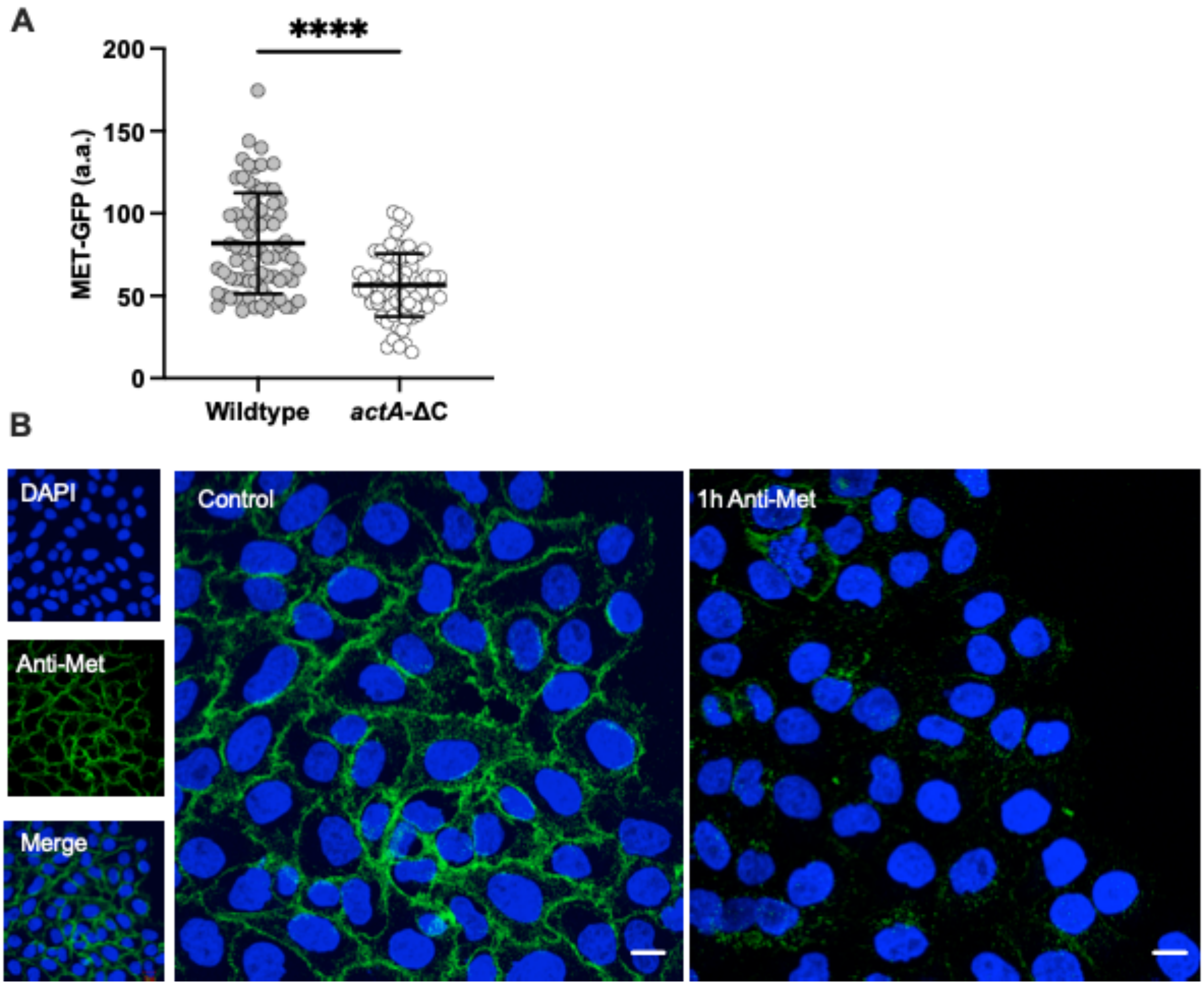

Figure S4. Analysis of MET clustering and expression. A) Analysis of MET clustering in data from Fig. 4C. Shown is the average intensity of MET-GFP staining associated with wild-type aggregates or *actA*-ΔC bacteria. 75 objects representative of three independent experiments depicted in circles, in black mean and standard deviation. Statistical analysis was performed using Mann-Whitney test (**** = $p < 0.0001$), normality assessed using Shapiro-Wilk test. **B)** Microscopy images of MET expression in HeLa cells. Shown are control cells stained with anti-MET antibody (left, in green) and cells pre-treated with anti-MET antibody for 1 h before staining (right). DAPI staining shown in blue. Images representative of three biological replicates. Scale bar 10 µm. On the left are individual channels as well as composite channel.

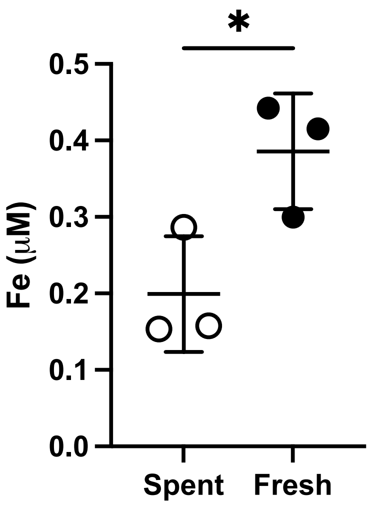

Figure S5. Depletion of iron concentration in spent media. Shown is iron concentration (μM) in fresh and spent media samples depicted as circles with mean and standard deviation based on three replicates. Statistical analysis was performed using t-test (* = $p < 0.05$), normality assessed with a Shapiro-Wilk test.
